# Supplementary material for: grandR: a comprehensive package for nucleotide conversion RNA-seq data analysis
Source: Nat Commun. 2023 Jun 15;14:3559. doi: 10.1038/s41467-023-39163-4 (PMC10272207; doi:10.1038/s41467-023-39163-4)
Supplement: Supplementary file 1 — Supplementary Information [file 41467_2023_39163_MOESM1_ESM.pdf]

grandR: a comprehensive package for nucleotide conversion RNA-seq data analysis

Supplementary Information

Teresa Rummel[1], Lygeri Sakellaridi[1], Florian Erhard[1,2]

1 Institute for Virology and Immunobiology, University of Würzburg, Versbacher Str. 7, 97078 Würzburg, Germany

2 Faculty for Informatics and Data Science, University of Regensburg, Bajuwarenstr. 4, 93053 Regensburg, Germany

Supplementary Figure 1: Quality control using grandR.

a Gene set enrichment analysis of MSigDB hallmark pathways. All pathways with adjusted P values < 5% (Benjamini-Hochberg adjusted for multiple testing) are shown.

b Vulcano plot of differentially expressed genes on total RNA level. The y axis shows the DESeq2 P value (two-sided Wald test) adjusted for multiple testing (Benjamini-Hochberg; FDR, false discovery rate). The numbers of genes above and below 5% FDR and for a threshold of 2-fold up- or downregulation are indicated.

c Heatmap of the 4sU-pulse chase experiment showing $\log_2$ fold changes vs the mean of the three 4sU naïve control samples (no4sU) of all n=7,215 genes. For a frame of reference, the genes from the heatmap in Figure 2b are included at the bottom. The color scale is the same as in Figure 2b and was chosen to show genes with less than 1.3-fold regulation in pure white.

d Principal component analysis of the pulse-chase data including the unlabeled control (no4sU), and all chase time points. This is similar to Figure 2a, which only shows the no4sU and the 0h chase time point (which has been labeled for 24h).

e Heatmap of the two 4sU-pulse experiments investigating the effect of m6A and miRNAs on RNA half-lives. Only data from wild-type cells are shown. Colors show $\log_2$ fold changes vs the mean of the three respective 4sU naïve control samples (no4sU) of all n=7,215 genes. The color scale was chosen to show genes with less than 1.3-fold regulation in pure white.

f Scatterplot showing half-lives computed using the non-linear least squares method for the untreated mock samples from the NXF1 data set for each gene. The x axis shows the half-life values considering the full time course (0h,2h,4h,8h), whereas the y axis shows the half-life values after excluding the 8h time point.

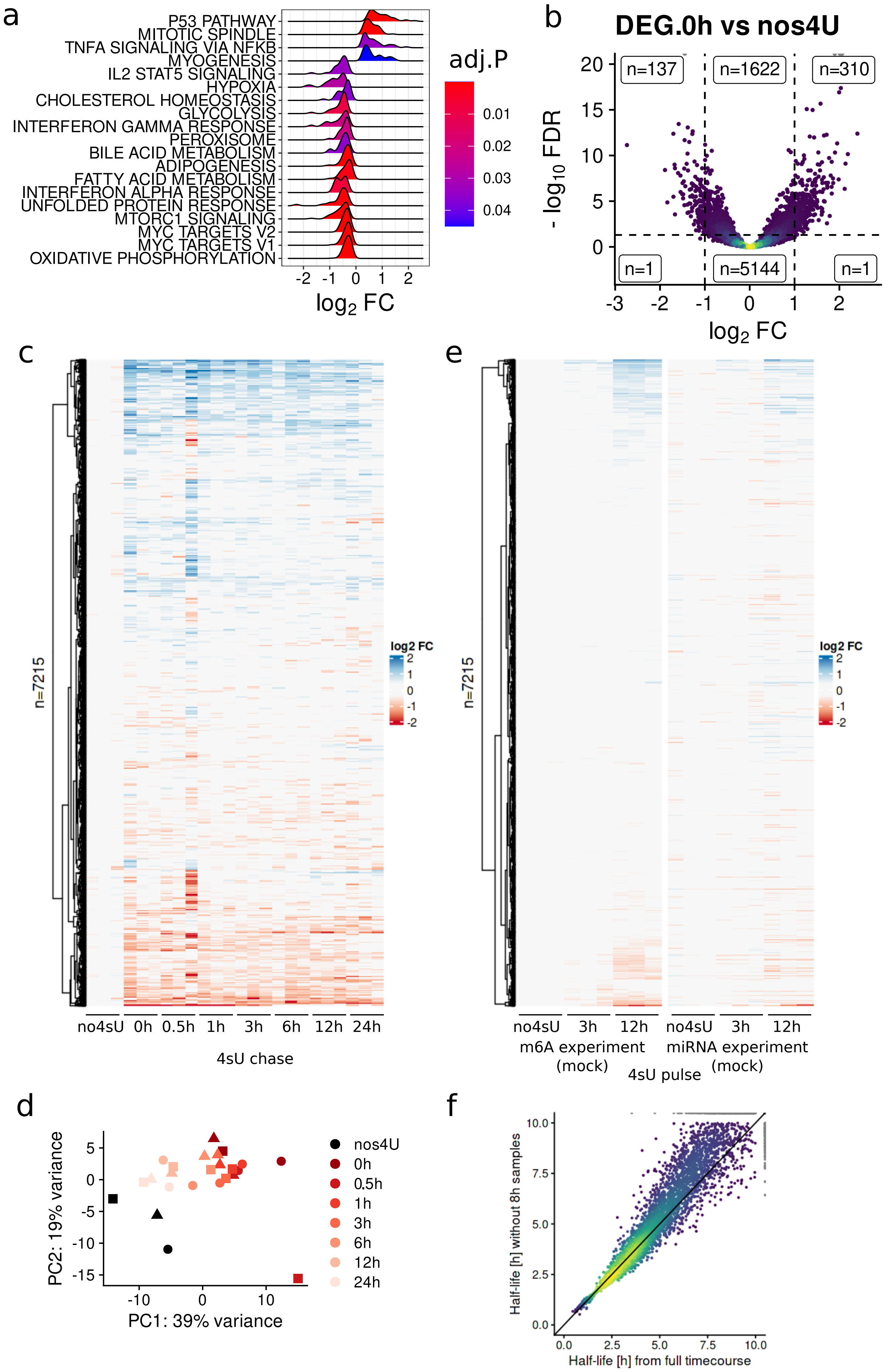
a
P53 PATHWAY
MITOTIC SPINDLE
TNFA SIGNALING VIA NFKB
MYOGENESIS
IL2 STAT5 SIGNALING
HYPOXIA
CHOLESTEROL HOMEOSTASIS
GLYCOLYSIS
INTERFERON GAMMA RESPONSE
PEROXISOME
BILE ACID METABOLISM
ADIPOGENESIS
FATTY ACID METABOLISM
INTERFERON ALPHA RESPONSE
UNFOLDED PROTEIN RESPONSE
MTORC1 SIGNALING
MYC TARGETS V2
MYC TARGETS V1
OXIDATIVE PHOSPHORYLATION
log2 FC
adj.P
b
DEG.0h vs nos4U
n=137
n=1622
n=310
n=1
n=5144
n=1
- log10 FDR
log2 FC
c
n=7215
log2 FC
no4sU 0h 0.5h 1h 3h 6h 12h 24h
4sU chase
e
n=7215
log2 FC
no4sU 3h 12h no4sU 3h 12h
m6A experiment (mock)
miRNA experiment (mock)
4sU pulse
d
PC2: 19% variance
PC1: 39% variance
nos4U
0h
0.5h
1h
3h
6h
12h
24h
f
Half-life [h] without 8h samples
Half-life [h] from full timecourse

Supplementary Figure 2: Evaluation of half-life estimates from simulated data

Half-lives and expression values were taken from the Mock samples of the SARS-CoV-2-data set. Timepoints 0h,1h,2h,4h,8h with 3 replicates each and 20 million reads per sample were simulated. Scatterplots compare the true, simulated half-life value for each gene against the half-life value estimated by the linear model (**a** and **e**), the non-linear least squares approach (**b** and **f**), the pulseR method (**c** and **g**) and the Bayesian approach (**d** and **h**). Results for simulated steady state gene expression (**a-d**) and when gene expression at 0h was perturbed (**e-h**) are shown. The Pearson correlation coefficient and associated P values (two-sided t test) are indicated.

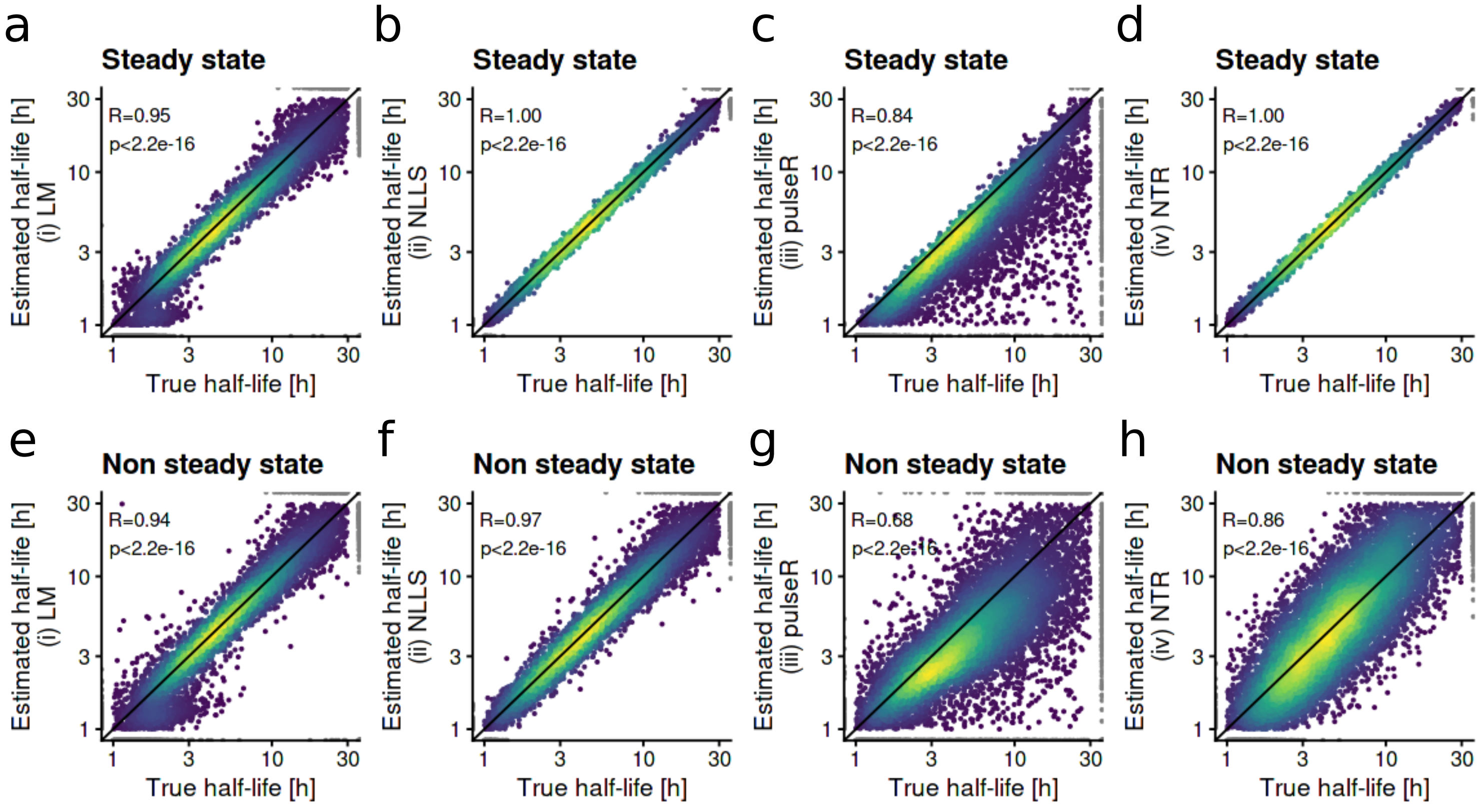

Supplementary Figure 3: Estimating half-lives using progressive labeling experiments.

a Boxplots showing the sizes of 95% half-life confidence intervals (CI; for LM and NLLS) or 95% half-life credible intervals (for NTR; center line, median; box limits, upper and lower quartiles; whiskers, 1.5x interquartile range; points, outliers). Simulations were performed under steady state conditions where the initial value was $a_0 \neq \sigma/\delta$ for each gene. Distributions for genes having the ground-truth inside or outside of the estimated CI are shown separately and the numbers of these genes are indicated. NTR represents the χ^2 approximation of CIs, NTR (exact) represents exact CIs computed numerically.

b Boxplots showing log2 fold changes of half-lives estimated by the NLLS method vs the ground truth of simulated data under steady state conditions. The distributions for different half-life classes are shown for several experimental settings involving the indicated number of replicates and time points (0-1h, n=204 genes; 1-2h, n=1,417 genes; 2-3h, n=1,686 genes; 3-4h, n=1,310 genes; 4-5h, n=1,108 genes; 5-6h, n=832 genes; 6-7h, n=639 genes; 7-8h, n=543 genes; >8h, n=3,096 genes; center line, median; box limits, upper and lower quartiles; whiskers, 1.5x interquartile range; points, outliers). **** indicate a p value of <0.001, ns indicate a p value >0.05; one-sided Brown-Forsythe test; exact p values for 0-1h, $p=2.83\times10^{-6}$; for 1-2h, $p=2.37\times10^{-8}$.

c Boxplots showing log2 fold changes of half-lives estimated by the NLLS method vs the ground truth of simulated data under steady state conditions. The distributions for different half-life classes are shown for several experimental settings involving the indicated number of replicates and time points (0-1h, n=204 genes; 1-2h, n=1,417 genes; 2-4h, n=2,996 genes; 4-8h, n=3,122 genes; >8h, n=3,096 genes; center line, median; box limits, upper and lower quartiles; whiskers, 1.5x interquartile range; points, outliers).

d Boxplots showing log2 fold changes of half-lives estimated by the NLLS method vs the ground truth of simulated data under steady state conditions for a full progressive labeling time course (1h,2h,4h and 8h). The distributions involving the indicated number of replicates and sequencing depth in million (M) reads are shown (n=10,835 genes; center line, median; box limits, upper and lower quartiles; whiskers, 1.5x interquartile range; points, outliers).

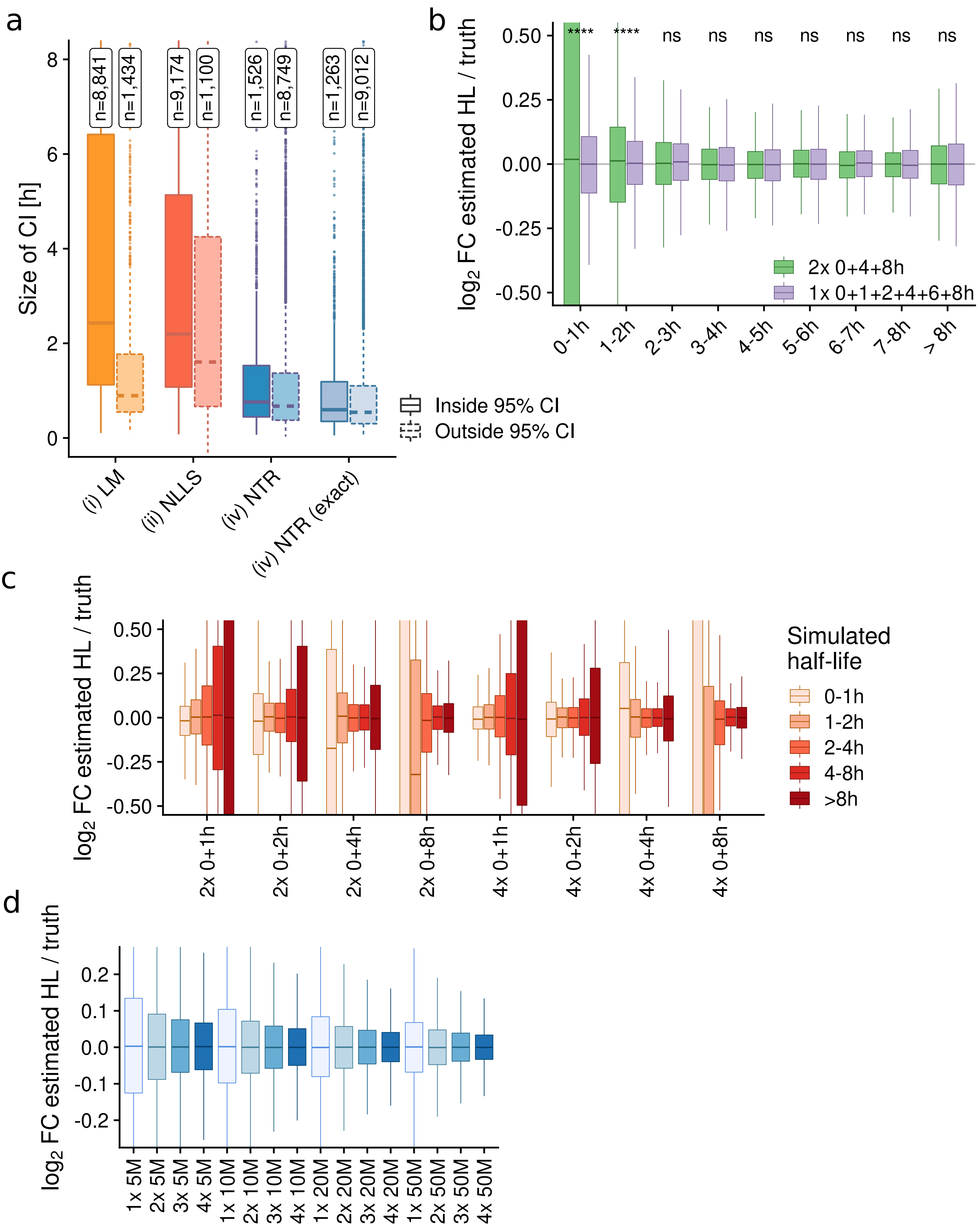
a
Size of CI [h]
n=8,841
n=1,434
n=9,174
n=1,100
n=1,526
n=8,749
n=1,263
n=9,012
(i) LM
(ii) NLLS
(iv) NTR
(iv) NTR (exact)
Inside 95% CI
Outside 95% CI
b
log2 FC estimated HL / truth
****
****
ns
2x 0+4+8h
1x 0+1+2+4+6+8h
0-1h
1-2h
2-3h
3-4h
4-5h
5-6h
6-7h
7-8h
> 8h
c
Simulated half-life
0-1h
1-2h
2-4h
4-8h
>8h
2x 0+1h
2x 0+2h
2x 0+4h
2x 0+8h
4x 0+1h
4x 0+2h
4x 0+4h
4x 0+8h
d
1x 5M
2x 5M
3x 5M
4x 5M
1x 10M
2x 10M
3x 10M
4x 10M
1x 20M
2x 20M
3x 20M
4x 20M
1x 50M
2x 50M
3x 50M
4x 50M

Supplementary Figure 4: Temporal recalibration of simulated data.

a Plot comparing labeling times before and after recalibration for simulated data. Data was simulated based on SARS-CoV-2-data (nominal labeling times 0h,1h,2h,4h,8h) either under steady state conditions or non-steady state conditions as indicated. For the 1-4h time points, an ineffective time of labeling as indicated was subtracted from the nominal times before simulation. Arrows show the $\log_2$ fold change of uncalibrated, nominal labeling times vs the true effective labeling time (start of the arrow) and of the recalibrated labeling time vs the true effective labeling time (tip of the arrow). Three replicates are shown by colors.

b Boxplots showing $\log_2$ fold changes of estimated (NLLS) half-lives vs. ground truth for n=9,162 genes before recalibration (uncalibrated), after recalibration (calibrated) and when the true effective labeling times were used (truth; center line, median; box limits, upper and lower quartiles; whiskers, 1.5x interquartile range; points, outliers).

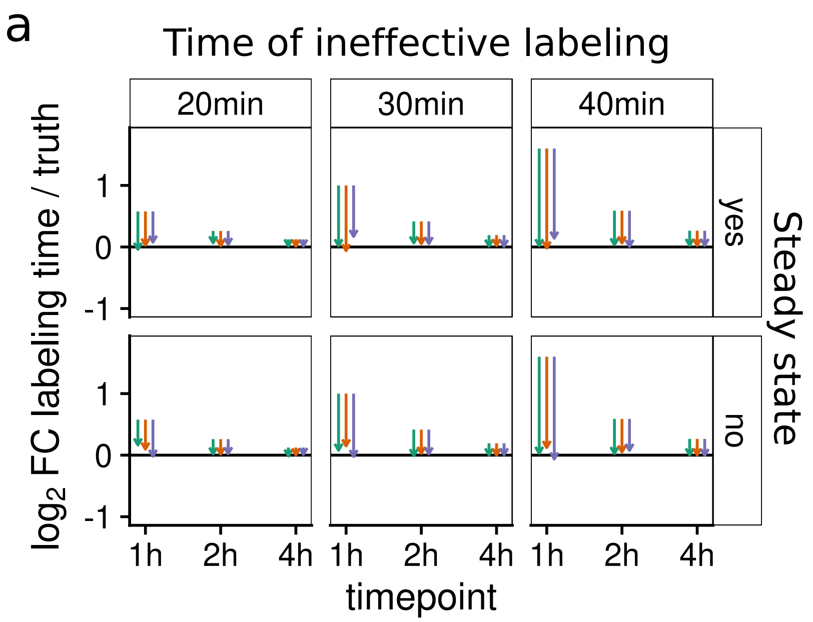

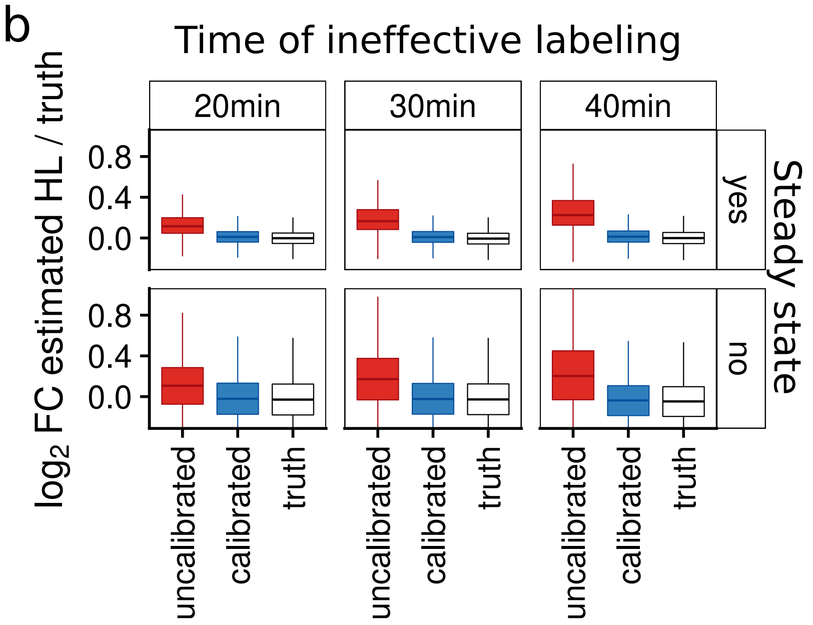

Supplementary Figure 5: Temporal recalibration of BANP depletion experiments.

Violin plots showing the distribution of estimated RNA half-lives (**a**) and synthesis rates (**b**) for n=11,096 genes estimated by our Bayesian hierarchical model for each experimental time point after temporal recalibration.

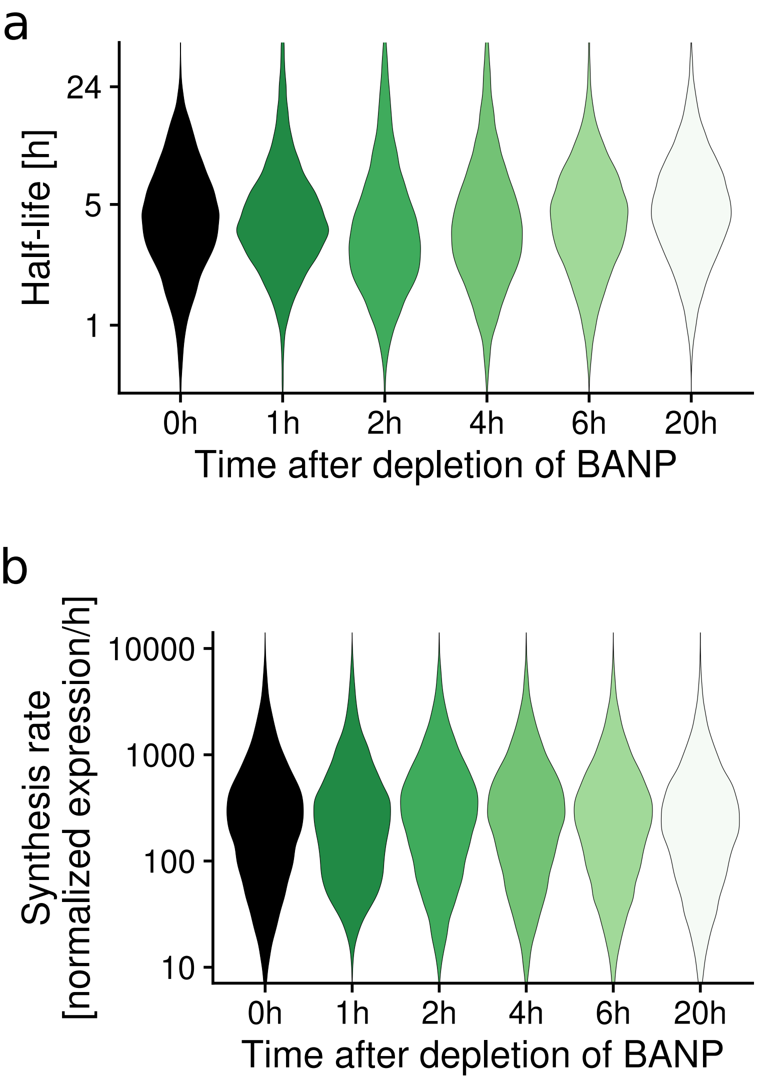

Supplementary Note 1

Contents

1 Non-steady state conditions vs non-constant rates 1
1.1 Constant rate model 1
1.2 Non-constant rate model 2

2 Bias of the constant rate approximation under non-constant rate conditions 2
2.1 Non-constant degradation rates 3
2.2 Non-constant synthesis rates 5

3 Appendix 6
3.1 Choices for $\sigma(t)$ and $\delta(t)$ 6
3.2 Proof that the estimated δ is the average of the initial and final degradation rates 6

1 Non-steady state conditions vs non-constant rates

1.1 Constant rate model

grandR is based on a model for RNA abundance $a(t)$ at time t assuming constant kinetic rates of RNA synthesis σ and degradation δ during the time of labeling:

$$\frac{da}{dt} = \sigma - \delta a(t) \tag{1}$$

This ordinary differential equation can analytically be solved for the boundary condition $a(0) = a_0$:

$$a(t) = \left(a_0 - \frac{\sigma}{\delta}\right) e^{-\delta t} + \frac{\sigma}{\delta} \tag{2}$$

If synthesis and degradation is in equilibrium, i.e. there is no change in RNA abundance $\frac{da}{dt} = 0$ (then $a(t) = \frac{\sigma}{\delta}$), a gene is said to be expressed at *steady state*.

It is important to stress that constant rates during the time of labeling do not imply steady state gene expression. For instance, a gene X might have an RNA synthesis rate of $\sigma_0 = 100$ and an RNA degradation rate $\delta = 0.25$ and is expressed at steady state $a(0) = 400$ before the start of labeling. Now let's say the synthesis rate is decreased to $\sigma = 50$ at time $t = 0$ (e.g. because a transcription factor that is important for the full activity of gene X is inactivated), and labeling is initiated at $t = 0$ for 2h. The RNA abundance $a(t)$ slowly approaches the new steady state level $\frac{50}{0.25} = 200$ for this gene (see Figure 1). Importantly, even if both rates are constant after time 0, the abundance changes significantly during the first hours.

Equation (2) implies a very specific shape of the temporal behavior of the RNA abundance: The time it takes for going half the distance to the new steady state is a constant. This constant is called the half-life and can be computed as $t_{1/2} = \frac{\log(2)}{\delta}$. For gene X, $t_{1/2} = 2.77$, which indeed is the time it takes for going from abundance $a(0) = 400$ to abundance $a(t_{1/2}) = 300$, i.e. half the way to the new steady state level $\frac{\sigma}{\delta} = 200$ (see Figure 1). We call this temporal behavior *half-life kinetics*.

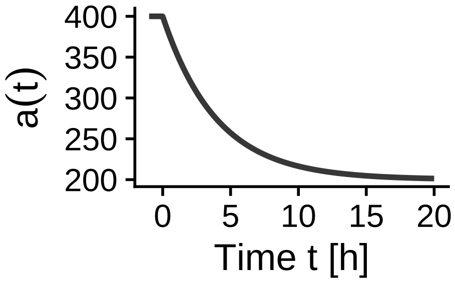

Figure 1: Temporal behavior of RNA abundance for a gene where the synthesis rate is decreased by half at time 0 (but constant for t>0).

1.2 Non-constant rate model

The constant rate model defined in equation (1) can be easily be generalized to non-constant rates:

$$\frac{da}{dt} = \sigma(t) - \delta(t)a(t) \tag{3}$$

Note that now both rates $\sigma(t)$ and $\delta(t)$ are functions of the time t. The analytical solution for this generalization has a more complicated form:

$$a(t) = a_0 \cdot e^{-d(0,t)} + \int_0^t e^{-d(\tau,t)}\sigma(\tau)d\tau \tag{4}$$

Here, $d(a,b) = \int_a^b \delta(\tau)d\tau$. If, in our example, the transcription factor for gene X is not instantaneously inactivated such that $\sigma = 50$ at $t = 0$, but σ linearly decreases over 20h from $a(0) = 100$ down to $a(20) = 50$ (see Figure 2A), the RNA abundance does not follow half-life kinetics (see Figure 2B)

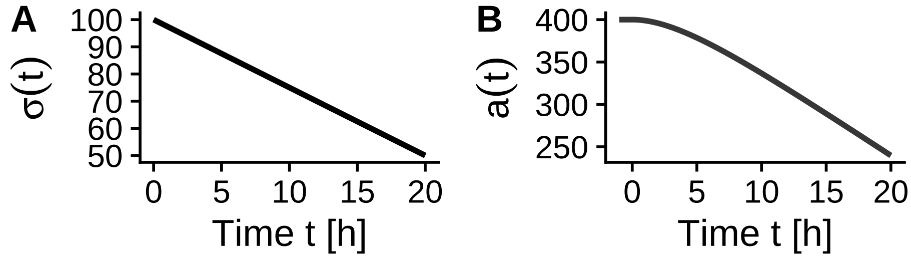

Figure 2: (A) Temporal behavior of a linearly decreasing synthesis rate. (B) Temporal behavior of the RNA abundance for a gene where the synthesis rate decrease as in A

2 Bias of the constant rate approximation under non-constant rate conditions

In many situations and experimental settings the assumption of constant rates is valid. For instance, if a transcription factor is depleted using an auxin inducible degron system, its protein levels are often not detectable anymore after a short period (e.g. 1h). During that short period the synthesis rate of each of its target genes decreases towards a new value σ but is expected to stay constant once the transcription factor has vanished completely. Thus, if labeling is initiated after that period, the constant rate assumption likely is valid.

However, it might not be possible to design an experiment where the rates of target genes can be assumed to be constant during the labeling time. In this case, the estimates of grandR are approximations and intuitively represent averages of these rates over the labeling time. In the following sections, we investigate the nature of these approximations more closely.

2.1 Non-constant degradation rates

First, we investigate a situation, where the degradation rate increases by a factor of 2 over 2h (Figure 3), i.e. where the RNA for a gene is increasingly destabilized.

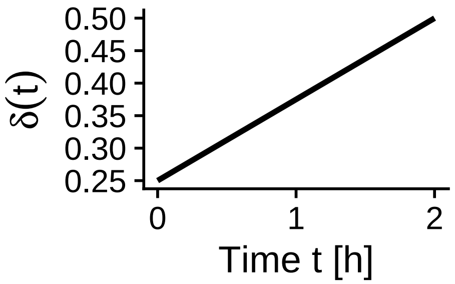

Figure 3: Temporal behavior of a linearly increasing degradation rate.

If labeling is initiated at time $t = 0$ for 2h (i.e. over the time shown in Figure 3), the RNA abundance between time 0 and 2h is a concave function (see Figure 4A). By contrast, if the degradation rate instantaneously increases to 0.5 at $t = 0$ (as it is assumed with constant rates), the RNA abundance follows half-life kinetics, i.e. is a convex function (see Figure 4B).

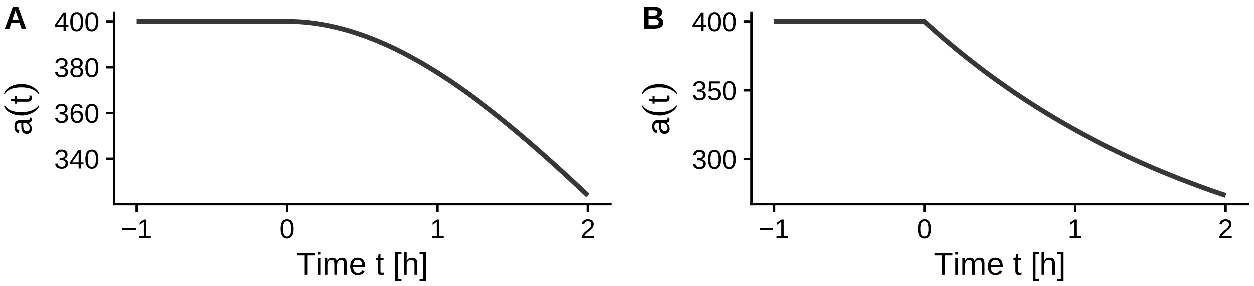

Figure 4: (A) Temporal behavior of the RNA abundance for a gene where the degradation rate linearly increases until it is doubled after 2h. (B) Temporal behavior of the RNA abundance for a gene where the degradation rate is abruptly doubled at t=0

For both situations, a slowly increasing degradation rate (case A), and a constant degradation rate (case B), the model in grandR can be used to estimate the degradation rate from data. In the former case (A), the degradation rate only is an approximation, in the latter case (B), the degradation rate is exact. Without adding noise (e.g.by simulating sequencing and 4sU conversion), the computed degradation rate for (B) indeed is 0.5. For (A) the computed degradation rate is 0.375, which is exactly in the middle of the initial degradation rate 0.25 and the final degradation rate 0.5 (see Figure 5A). It can indeed be proven (see Appendix below) that the approximation for linearly increasing or decreasing degradation rates is exactly the mean of the initial and final degradation rates. However, if the increase is not linear, but accelerates (see Figure 5B) or decelerates over time (see Figure 5C), the approximation is smaller or greater than the mean of the initial and final degradation rates, respectively.

The approximation can be considered a weighted mean: In our example of an accelerating function $\delta(t) = 0.25 + 0.0625 \cdot t^2$, the degradation rate is in between 0.25 and 0.3125 during the first hour (a relatively small interval), and in between 0.3125 and 0.5 during the second hour (a much larger interval, see Figure 5A). The approximation here is $\delta = 0.333$, i.e. *below* the mean of initial and final rates (which would be 0.375).

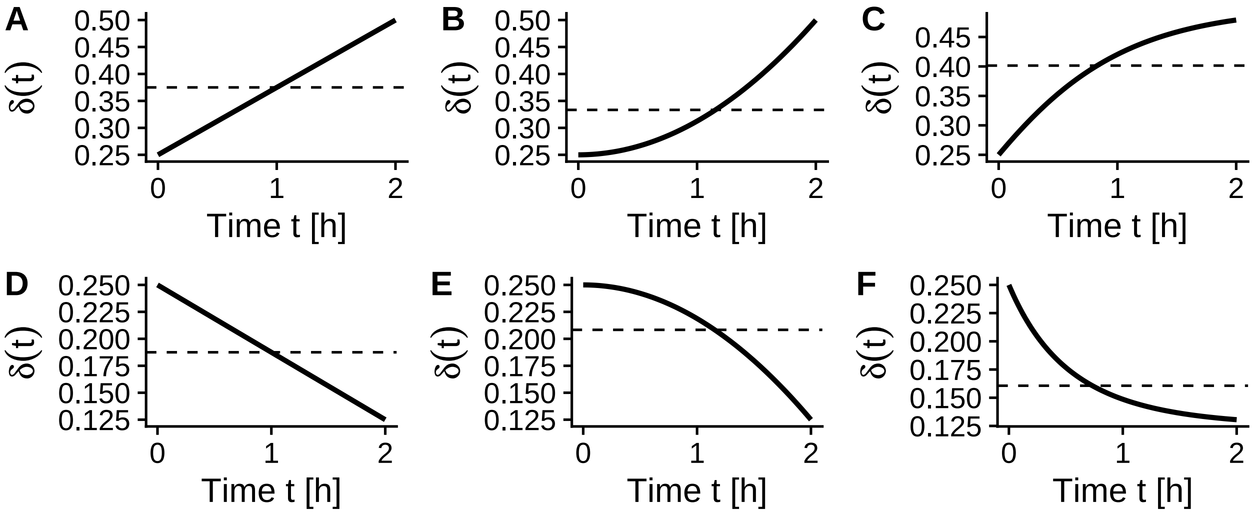

Figure 5: (A) Temporal behavior of a linearly increasing degradation rate with the degradation rate estimated using the constant rate approximation shown as a dashed line. (B-C) Same as A, but here the degradation rate does not increase linearly, but the increase either accelerates (B) or slows down (C) over time. See the appendix for a description of these functions. (D-F) The same as in A-C, but with decreasing degradation rates as indicated.

Thus, the approximation respects that $\delta(t)$ spends the first hour at smaller values. The exact nature of this weighting down or up depends on the shape of the degradation rate function. With decreasing degradation rates equivalent down or up weighting can be observed (see Figure 5D-F).

In summary, the constant rate approximation provides estimates of non-constant degradation rates that correspond to a weighted average of the degradation rates during the labeling time. If the degradation rate function $\delta(t)$ is indeed linear, the estimated degradation rate is exactly the mean of the initial and final degradation rates.

2.2 Non-constant synthesis rates

For non-constant synthesis rates, a similar picture can be observed as for degradation rates (see Figure 6), where non-constant synthesis rates are approximated by a weighted average of the synthesis rates during the labeling time. Importantly, however, now even for $\sigma(t)$ being a linear function, the approximation is not exactly the mean of the initial and final synthesis rates (see Figure 6A). For instance, with simulated $\sigma(0) = 100$, $\sigma(2) = 200$, and a constant $\delta(t) = 0.25$, the approximated synthesis rate is $\hat{\sigma} = 154.1$, which is slightly larger than the average $\sigma(1) = 150$.

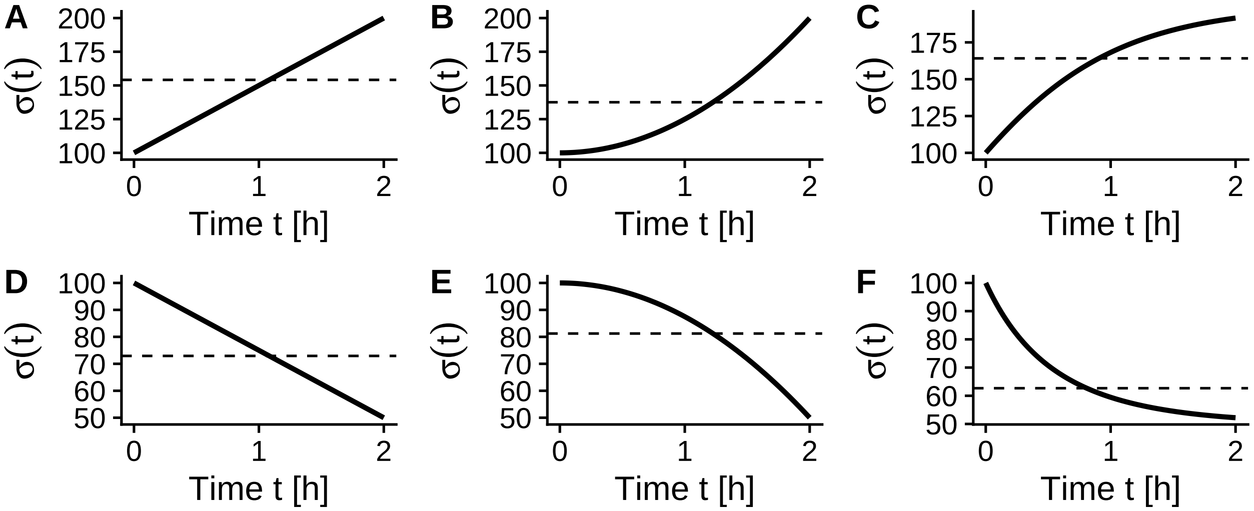

Figure 6: (A) Temporal behavior of a linearly increasing synthesis rate with the synthesis rate estimated using the constant rate approximation shown as a dashed line. (B-C) Same as A, but here the synthesis rate does not increase linearly, but the increase either accelerates (B) or slows down (C) over time. See the appendix for a description of these functions. (D-F) The same as in A-C, but with decreasing synthesis rates as indicated.

Interestingly, while for non-constant degradation rates the approximation is independent on the synthesis rate, for non-constant synthesis rates, the approximation is indeed dependent on the degradation rate: With low degradation rates (e.g. $\delta(t) = 0.25$ as above), the estimated synthesis rate is very close to the mean of the initial and final synthesis rates. However, with much larger degradation rates (e.g. such as $\delta(t) = 5$), the estimated synthesis rates approach the final synthesis rate (see Figure 7). A degradation rate of $\delta(t) = 5$ corresponds to an RNA half-life of $t_{1/2} = 8.3$ min. Thus, it is not surprising that the estimated synthesis rate is much closer to the final synthesis rate $\sigma(2)$, since most of the RNA that is present at the end of labeling is not older than a few minutes. Indeed for small degradation rates that correspond to RNA half-lives larger than the 2h of labeling, the estimated synthesis rates are close to the average of the initial and final synthesis rates (see Figure 7C).

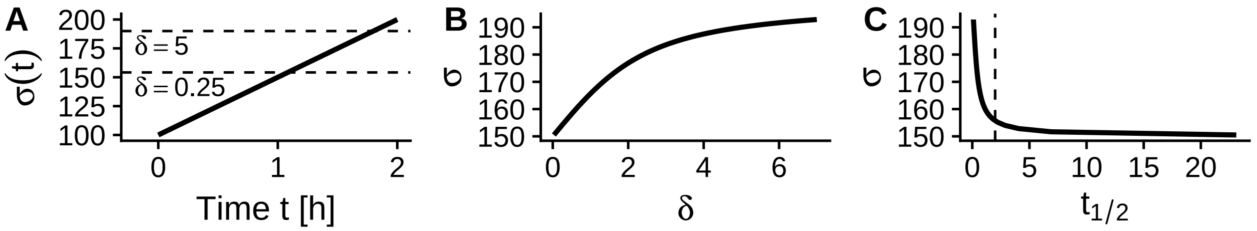

Figure 7: (A) Temporal behavior of a linearly increasing synthesis rate with the synthesis rates estimated using the constant rate approximation for different degradation rates shown as dashed lines. (B) For different degradation rates (x axis), the synthesis rates estimated using constant rate approximation are shown (y axis). (C) The same as in B, but now the x axis shows the corresponding RNA half-lives instead of degradation rates. The time of labeling (2h) is indicated by a dashed line.

In summary, the constant rate approximation provides estimates of non-constant synthesis rates that correspond to a weighted average of the synthesis rates during the labeling time. If the synthesis rate function $\sigma(t)$ is linear and the degradation of new RNA is negligible, the estimated synthesis rate is close to the mean of the initial and final synthesis rates.

3 Appendix

3.1 Choices for $\sigma(t)$ and $\delta(t)$

Depending on the biological system under study, the rate functions $\delta(t)$ and $\sigma(t)$ might exhibit various shapes. Here, we studied two different functions:

$$f_1(t) = s + \frac{e-s}{v^p} \cdot t^p \tag{5}$$

$$f_2(t) = s \cdot c^{1-e^{-td}} \tag{6}$$

For both functions, s is the initial value (i.e., $f_1(0) = f_2(0) = s$). For f_1, e is the final value at time v (i.e., $f_1(v) = e$), and $p \geq 1$ determines the degree of acceleration with $p = 1$ resulting in a linear function (see Figures 5A, 5D, 6A and 6D), and $p = 2$ has been used as our example for an accelerating function (see Figures 5B, 5E, 6B and 6E).

By contrast f_2 represents a saturation curve that approaches $\lim_{t\to\infty} f_2(t) = s \cdot c$, i.e. the constant c indicates a fold change. The speed of the saturation curve approaching the limit is determined by d. Here we used f_2 for our examples of decelerating rates (see Figures 5C, 5F, 6C and 6F). We used $d = 1.39$ meaning that half the way to the limit is achieved after a quarter of the full time.

3.2 Proof that the estimated δ is the average of the initial and final degradation rates

For a linear degradation rate function $\delta(t) = a + b \cdot t$ and a constant synthesis rate $\sigma(t) = \sigma$, equation (4) becomes:

$$
\begin{aligned}
a(t) &= a_0 \cdot e^{-d(0,t)} + \sigma \int_0^t e^{-d(\tau,t)} d\tau & (7)\\
&= a_0 \cdot e^{-d(0,t)} + \sigma \cdot e^{-d(0,t)} \int_0^t e^{d(0,\tau)} d\tau & (8)\\
&= e^{-d(0,t)} \cdot \left(a_0 + \sigma \int_0^t e^{d(0,\tau)} d\tau \right) & (9)\\
& & (10)
\end{aligned}
$$

Here, $d(0,t) = \int_0^t \delta(\tau) d\tau$ can be simplified to $d(0,t) = t \cdot (a + b\frac{t}{2}) = t \cdot \delta(\frac{t}{2})$. For estimating δ in grandR, first the NTR is computed from the abundance of new RNA $a_{new}(t)$ and $a(t)$:

$$
\begin{aligned}
NTR &= \frac{a_{new}(t)}{a(t)} & (11)\\
&= \frac{\sigma \int_0^t e^{d(0,\tau)} d\tau}{a_0 + \sigma \int_0^t e^{d(0,\tau)} d\tau} & (12)
\end{aligned}
$$

The degradation rate is computed from this as

$$
\begin{aligned}
\hat{\delta} &= -\frac{1}{t} \log \left(\frac{a(t)}{a_0} (1 - NTR) \right) & (13)\\
&= -\frac{1}{t} \log \left(\frac{a(t)}{a_0} \frac{a_0}{a_0 + \sigma \int_0^t e^{d(0,\tau)} d\tau} \right) & (14)\\
&= -\frac{1}{t} \log \left(\frac{e^{-d(0,t)} \cdot \left(a_0 + \sigma \int_0^t e^{d(0,\tau)} d\tau \right)}{a_0 + \sigma \int_0^t e^{d(0,\tau)} d\tau} \right) & (15)\\
&= -\frac{1}{t} \left(-d(0,t) \right) & (16)\\
&= a + b\frac{t}{2} & (17)
\end{aligned}
$$

Thus, the approximation of the degradation rate is the mean of the initial and final degradation rates $\frac{1}{2}(\delta(0) + \delta(2)) = \frac{1}{2}(a + a + b \cdot t) = a + b\frac{t}{2}$.
