# Supplementary material for: grandR: a comprehensive package for nucleotide conversion RNA-seq data analysis
Source: Nat Commun. 2023 Jun 15;14:3559. doi: 10.1038/s41467-023-39163-4 (PMC10272207; doi:10.1038/s41467-023-39163-4)
Supplement: Supplementary file 3 — Description of Additional Supplementary Files [file 41467_2023_39163_MOESM3_ESM.pdf]

Description of Additional Supplementary Files

File Name: Supplementary Data 1
Description: Differential gene expression table for the mESC pulse-chase data.

File Name: Supplementary Software 1
Description: R notebooks reproducing all analyses. These and pre-computed simulated data sets are also available at https://doi.org/10.5281/zenodo.7843048.
